# A Residual Amplitude Modulation Noise Suppression Method Based on Multi-Harmonic Component Decoupling

**DOI:** 10.3390/s26061841

**Published:** 2026-03-14

**Authors:** Qiwu Luo, Hang Su, Yibo Wang, Chunhua Yang

**Affiliations:** School of Automation, Central South University, Changsha 410083, China; luoqiwu@csu.edu.cn (Q.L.); 244611053@csu.edu.cn (H.S.); ychh@csu.edu.cn (C.Y.)

**Keywords:** residual amplitude modulation, lateral peak inclination angle, multiple harmonic component decoupling

## Abstract

Wavelength modulation spectroscopy (WMS) is a representative implementation of tunable diode laser absorption spectroscopy (TDLAS), enabling reliable gas component analysis with concentration-related information derived from harmonic component extraction, while offering enhanced noise immunity for trace gas sensing in open environments. However, due to the strong coupling between laser wavelength and intensity, wavelength modulation inevitably introduces residual amplitude modulation (RAM), which significantly degrades measurement accuracy. To address this issue, this study introduces a RAM suppression algorithm based on multiple harmonic component decoupling (MHCD), using the second-harmonic lateral peak inclination angle (LPIA) as a characteristic indicator. Unit harmonic operators for the first, second, and third harmonics are designed, and an original harmonic reconstruction model is established via linear superposition of harmonic components. The optimal harmonic component ratio is determined at the composite operator with the maximum cross-correlation coefficient, and RAM noise is eliminated through a multi-harmonic decoupling matrix. Repetitive measurements on 22 mm pharmaceutical vials with 4% oxygen concentration demonstrate that MHCD reduces the second-harmonic LPIA from 18.07° to 8.56°. Concentration discrimination experiments conducted on seven groups of 22 mm vials with 2% concentration steps (0–12%) show that MHCD increases the true positive rate by 6–11% and decreases the false positive rate by 4–9%, confirming its effectiveness for pharmaceutical online inspection applications.

## 1. Introduction

Tunable diode laser absorption spectroscopy (TDLAS) is a widely adopted gas-sensing technique known for its high sensitivity [1], rapid response [2], and non-contact measurement capability [3]. These advantages have enabled its extensive application in pharmaceutical manufacturing equipment, steel and metallurgical processes, as well as energy and environmental monitoring systems [4,5,6]. As a representative application, for evaluating the sealing integrity of pharmaceutical vials, an ultra-narrowband laser operating near the strong oxygen absorption feature at 760.88 nm can be used to probe the headspace of glass containers. This approach enables nondestructive, in situ, and rapid quantification of residual oxygen content, offering significant potential for high-speed and flexible sterile-product manufacturing lines [7,8,9].

TDLAS techniques generally include direct absorption spectroscopy (DAS) and wavelength-modulation spectroscopy (WMS). Owing to its ability to shift the laser frequency into a higher and narrower modulation band, WMS exhibits enhanced suppression of environmental disturbances and therefore holds greater potential for trace-gas measurements in open and complex industrial settings [10,11,12]. In a typical WMS system, the laser driving current is modulated by superimposing a low-frequency sawtooth waveform with a high-frequency sinusoidal signal. This current modulation not only induces wavelength modulation but also inevitably alters the output intensity of the laser, leading to undesired intensity modulation. When this intensity modulation is not fully eliminated or interacts with the wavelength modulation, a residual amplitude modulation component arises [13,14,15]. RAM distorts the spectral line shape of the detected signal, causing the amplitude and morphology of harmonic components—such as the second harmonic—to deviate from their ideal forms. This distortion degrades the accuracy of absorption-line analysis and ultimately compromises concentration inversion [16,17]. Furthermore, RAM introduces additional noise and reduces the system’s signal-to-noise ratio, thereby impairing sensitivity and measurement precision. The issue becomes particularly critical in trace-gas detection, where the inherently weak absorption features may be completely masked by RAM-induced noise, making it difficult to reliably identify or quantify the target species [18,19,20].

Existing studies on residual amplitude modulation noise suppression can be broadly categorized into two approaches. The first focuses on hardware optimization within the detection system, including optical paths and electronic circuits. For example, Chang et al. [21] compared subtraction and division pre-processing circuits, showing that division circuits are more effective in RAM suppression. Chakraborty et al. [22] employed fiber delay lines to maintain a 180° phase difference between dual optical paths, balancing amplitudes to cancel RAM. Upadhyay et al. [23] further integrated fiber ring resonators to monitor the phase difference between amplitude and frequency modulation, compensating for phase variations. Bao et al. [24] used adjustable optical attenuators to stabilize intensity variations, effectively suppressing modulation amplitude; however, their method is limited by the attenuator response time and is only effective at low modulation frequencies. Wei et al. [25] dynamically adjusted laser modulation coefficients to iteratively shape RAM-contaminated second-harmonic signals toward ideal waveforms, though the point-by-point adjustment is laborious and unstable. Zhang et al. [26] proposed a dual-channel lock-in amplifier architecture to monitor and eliminate RAM by tuning the reference phase difference, which significantly improves SNR but at increased hardware cost. These hardware-based approaches generally suffer from poor reproducibility, high cost, and limited applicability in short optical paths or spatially constrained scenarios. The second category includes algorithmic noise suppression and environmental calibration methods. Musialek et al. [27] applied genetic algorithms to optimize TDLAS/WMS modulation and harmonic detection parameters, reducing RAM and background noise at the system level and enhancing SNR. Chui et al. [28] applied the Gabor time–frequency transform to denoise second-harmonic signals, thereby reducing the coupling between RAM and other noise components and improving demodulation stability. Sun et al. [29] proposed a laser linewidth analysis combined with an adaptive variational mode decomposition–Savitzky–Golay (VMD–SG) filtering algorithm to suppress baseline noise fluctuations in raw detection signals. Zhao et al. [30] developed a hybrid denoising approach based on long short-term memory (LSTM) networks and denoising autoencoders (DAE), specifically designed for noise reduction in TDLAS second-harmonic signals. Lan et al. [31] introduced a logarithm-based background noise suppression method, which can theoretically mitigate RAM noise to a certain extent. While these algorithmic approaches avoid additional hardware cost, they typically target spectral signals directly without explicitly addressing the generation mechanisms of RAM, and thus may offer suboptimal suppression performance.

Building upon the aforementioned studies, this work proposes a residual amplitude modulation suppression approach based on the decoupling of multi-order harmonic components. The method constructs a composite harmonic operator from theoretically derived harmonic bases, which is then used to learn and estimate the proportional contributions of different harmonic orders under the influence of RAM. These estimated amplitude ratios are subsequently applied to decouple the multi-order harmonic contributions embedded in the distorted second-harmonic signal, thereby enabling the purification of the second harmonic. Compared with prior studies, the proposed strategy requires only post-processing of the measured spectral signal and does not necessitate any modifications to the existing optical or electronic hardware. This advantage provides greater system compactness and enhances engineering applicability. Moreover, the method is inherently independent of the frequency-modulation parameters and maintains stable compensation performance under arbitrary variations in intensity-modulation amplitude or phase. Consequently, the approach offers a flexible and low-cost RAM mitigation solution that is particularly well suited for space-constrained residual oxygen detection in pharmaceutical vials. Finally, a system for residual oxygen measurement in sealed vials was established, employing the oxygen absorption line centered at 760.884 nm for seal-integrity assessment. Using a standard test vial containing 0% oxygen as an example, the true-positive detection rate (TPR) improved from 86.5% to 94% after applying the proposed method, thereby validating its effectiveness and practical feasibility.

## 2. Background and Methodology

### 2.1. Principle of TDLAS-WMS

The detection principle of wavelength-modulation spectroscopy (WMS) is based on modulating the injection current of a tunable diode laser with a composite signal formed by superimposing a high-frequency sinusoidal waveform onto a low-frequency sawtooth waveform. Due to the approximately linear relationship between the injection current and the laser’s output optical frequency, this modulation scheme enables the laser frequency to be scanned over a predefined spectral range. The temporal response and the transfer characteristics between the laser drive current and the resulting output frequency can be described as follows [32,33]:

(1) $$i=\bar{i}+i_{m}\cos(\omega t)$$

(2) $$v=\bar{v}+v_{m}\cos(\omega t)$$

(3) v=ζi

In this formulation, $\bar{i}$ denotes the mean value of the sawtooth-modulated injection current; $i_{m}$ is the amplitude of the sinusoidal current-modulation component; ω represents the angular frequency of the sinusoidal modulation; $\bar{v}$ denotes the center optical frequency corresponding to the sawtooth scanning range; $v_{m}$ is the amplitude of the frequency-modulation term; and ξ denotes the linear current-to-frequency conversion coefficient of the laser.

It should be noted that the modulation of the injection current simultaneously induces not only frequency modulation but also accompanying variations in the emitted optical intensity. As a result, the instantaneous output intensity of the laser, denoted as W/m^2^, can be expressed as:

(4) $$I_{0}(t)={\bar{I}}_{0}+\delta I_{m}\cos(\omega t+\phi )$$

In this context, ${\bar{I}}_{0}$ denotes the mean value of the laser-intensity scanning component, $\delta I_{m}$ represents the amplitude of the intensity-modulation term, and ϕ is the phase offset between the frequency-modulation signal and the corresponding intensity-modulation response. When the modulated laser beam propagates through the target gas, the transmitted intensity can be described according to the Beer–Lambert law. Accordingly, the instantaneous transmitted optical power is given by:

(5) $$I_{t}(t)=I_{0}(t)e^{-\alpha (v,T)}=I_{0}(t)e^{-PCLS(T)\varphi (v)}$$

where α(v,T) denotes the spectral absorbance per unit concentration and unit path length at temperature and frequency; P is the total gas pressure; S(T) represents the line strength of the absorption transition at temperature; φ(v) is the corresponding line-shape function; C is the concentration of the target species; and L is the effective absorption path length. Since the magnitude of α(v,T) is sufficiently small in conventional experimental environments such that higher-order absorption contributions can be neglected, Equation (5) may be asymptotically approximated as [34]:

(6) $$I_{t}(t)=({\bar{I}}_{0}+\delta I_{m}\cos(\omega t+\phi ))(1-PCLS(T)\varphi (v))$$

The absorption line shape in this paper is confined as Lorentzian profile, which Fourier-series expansion can be expressed as:

(7) $$\varphi (v)=\varphi (\bar{v}+\cos(\omega t))=\sum_{n=0}^{\infty }H_{n}\cos(n\omega t)$$

where $H_{n}$ denotes the N-th Fourier coefficient. Reid et al. [35] provided the general analytical form of $H_{n}$, which can be written as:

(8) $$H_{n}\left(x,m\right)=\frac{1}{2}\cdot \varepsilon_{n}\cdot i^{n}\cdot \frac{{\left\{{\left[{\left(1-ix\right)}^{2}+m^{2}\right]}^{1/2}-\left(1-ix\right)\right\}}^{n}}{m^{n}{\left[{\left(1-ix\right)}^{2}+m^{2}\right]}^{1/2}}+c.c$$

(9) $$\varepsilon_{0}=1,\varepsilon_{i}=2,\left(i=2,3,\ldots ,n\right)$$

(10) $$x=\frac{\nu \left(t\right)-\bar{\nu }}{\nu_{c}},m=\frac{\nu_{m}}{\nu_{c}}$$

In this expression, x denotes the normalized wavenumber, v(t) is the instantaneous optical frequency of the laser, $v_{c}$ represents the half width at half maximum (HWHM) of the Lorentzian line shape. The parameter m denotes the normalized frequency-modulation amplitude, commonly referred to as the modulation depth, c.c indicates the complex conjugate term.

Combine Equations (6) and (7), let ξ=PCLS(T), Equation (6) can be shown as:

(11) $$I_{t}\left(t\right)=\left({\bar{I}}_{0}+\delta I_{m}\cos\left(\omega t+\phi \right)\right)\left(1-\zeta \sum_{n=0}^{\infty }H_{n}\cos\left(n\omega t\right)\right)$$

This equation can be further expressed as:

(12) $$\begin{array}{l} I_{t}(t)={\bar{I}}_{0}+\delta I_{m}\cos\left(\omega t+\phi \right)-{\bar{I}}_{0}\zeta \sum_{n=0}^{\infty }H_{n}\cos\left(n\omega t\right)\\ -\delta I_{m}\zeta \sum_{n=0}^{\infty }H_{n}\cos\left(n\omega t\right)\cdot \cos\left(\omega t+\phi \right) \end{array}$$

By applying the product-to-sum identities, the infinite-series term in Equation (11) is further expanded and regrouped according to the N-th order frequency components into a Fourier-series representation. The instantaneous transmitted laser intensity is thereby expressed as follows:

(13) $$I_{t}\left(t\right)=D_{0}+\sum_{n=1}^{\infty }\left(A_{n}\sin\left(n\omega t\right)+B_{n}\cos\left(n\omega t\right)\right)$$

(14) $$\left\{\begin{array}{c} D_{0}={\bar{I}}_{0}-{\bar{I}}_{0}\zeta H_{0}\\ A_{1}=\delta I_{m}\cos\phi -{\bar{I}}_{0}\zeta H_{1}-\delta I_{m}\zeta \left(H_{0}+\frac{1}{2}H_{2}\right)\cos\phi \\ B_{1}=-\delta I_{m}\sin\phi -\delta I_{m}\zeta \left(\frac{1}{2}H_{2}-H_{0}\right)\sin\phi \\ A_{n}=-{\bar{I}}_{0}\zeta H_{n}-\frac{\delta I_{m}\zeta }{2}\left(H_{n-1}+H_{n+1}\right)\cos\phi \\ B_{n}=\frac{\delta I_{m}\zeta }{2}\left(H_{n-1}-H_{n+1}\right)\sin\phi \end{array}\right.$$

The Fourier coefficients $A_{n}$ in Equations (13) and (14) are directly proportional to the concentration of the gas and the effective absorption path length [35].

### 2.2. Harmonic Extraction and LPIA

In practical measurements, the harmonic components are typically extracted and recorded using a quadrature demodulation approach. The principle of quadrature demodulation involves multiplying the measured signal by a reference signal generated within the system itself, followed by low-pass filtering to obtain the orthogonal harmonic components. The N-th order reference signal used in quadrature demodulation can be expressed as [36]:

(15) $$\left\{\begin{array}{l} V_{ref\_I}=R_{n}\cos\left(n\omega t+\theta_{n}\right)\\ V_{ref\_Q}=R_{n}\sin\left(n\omega t+\theta_{n}\right) \end{array}\right.$$

where $R_{n}$ and $\theta_{n}$ denote the amplitude and phase of the reference signal, respectively. Based on the aforementioned principles, the expressions for the N-th order harmonic components can be derived. Taking the n-th harmonic as an example, the transmitted optical intensity is multiplied by the in-phase (I) and quadrature (Q) reference signals, yielding [37]:

(16) $$\begin{array}{cc} V_{I}=V_{ref\_I}\cdot I_{t}= & \frac{R_{n}A_{n}}{2}\left[\cos\left(2n\omega t+\theta_{n}\right)+\cos\theta_{n}\right]\\ & +\frac{R_{n}B_{n}}{2}\left[\sin\left(2n\omega t+\theta_{n}\right)-\sin\theta_{n}\right]+ο\left(\cos k\omega t\right) \end{array}$$

(17) $$\begin{array}{cc} V_{Q}=V_{reQ\_I}\cdot I_{t}= & \frac{R_{n}A_{n}}{2}\left[\cos\left(2n\omega t+\theta_{n}\right)+\sin\theta_{n}\right]\\ & -\frac{R_{n}B_{n}}{2}\left[\cos\left(2n\omega t+\theta_{n}\right)-\cos\theta_{n}\right]+ο\left(\cos k\omega t\right) \end{array}$$

Here, k≠0 and o(cos(kωt)) denotes the high-frequency components resulting from the multiplication. After passing the I- and Q-channel signals through low-pass filters, the high-frequency components are removed, leaving only the zero-frequency (DC) terms. Consequently, the final acquired signals can be expressed as follows:

(18) $$\left\{\begin{array}{c} V_{I\_filt}=\frac{R_{n}A_{n}}{2}\cos\theta_{n}-\frac{R_{n}B_{n}}{2}\sin\theta_{n}\\ V_{Q\_filt}=\frac{R_{n}A_{n}}{2}\sin\theta_{n}+\frac{R_{n}B_{n}}{2}\cos\theta_{n} \end{array}\right.$$

To eliminate the influence of the $\theta_{n}$ on the demodulated signals, it is common practice to compute the root of the sum of squares of the I- and Q-channel harmonic components. This procedure yields the absolute magnitude of the n-th harmonic, which can be calculated as follows:

(19) $$\begin{array}{cc} X_{nf} & =\sqrt{V_{I\_filt}^{2}+V_{Q\_filt}^{2}}\\ & =\sqrt{\begin{array}{l} \left(\frac{R_{n}^{2}A_{n}^{2}}{4}\sin^{2}\theta_{n}+\frac{R_{n}^{2}A_{n}B_{n}}{2}\sin\theta_{n}\cos\theta_{n}+\frac{R_{n}^{2}B_{n}^{2}}{4}\cos^{2}\theta_{n}\right)+\\ \left(\frac{R_{n}^{2}A_{n}^{2}}{4}\cos^{2}\theta_{n}-\frac{R_{n}^{2}A_{n}B_{n}}{2}\sin\theta_{n}\cos\theta_{n}+\frac{R_{n}^{2}B_{n}^{2}}{4}\sin^{2}\theta_{n}\right) \end{array}}\\ & =\frac{R}{2}\sqrt{A_{n}^{2}+B_{n}^{2}} \end{array}$$

In practical measurements, the quadrature-demodulated signal at twice the modulation frequency is typically employed as the basis for concentration retrieval, corresponding to the extraction of the second harmonic. The expression for this component is given as follows:

(20) $$X_{2f}=\frac{R}{2}\zeta \sqrt{{\bar{I}}_{0}H_{2}^{2}+\frac{\delta I_{m}^{2}}{4}\left(H_{1}^{2}+H_{3}^{2}\right)+\frac{\delta I_{m}^{2}}{2}H_{2}\left(H_{1}+H_{3}\right)\cos\phi +\frac{\delta I_{m}^{2}}{2}H_{1}H_{3}\cos2\phi }$$

**Figure 1:**
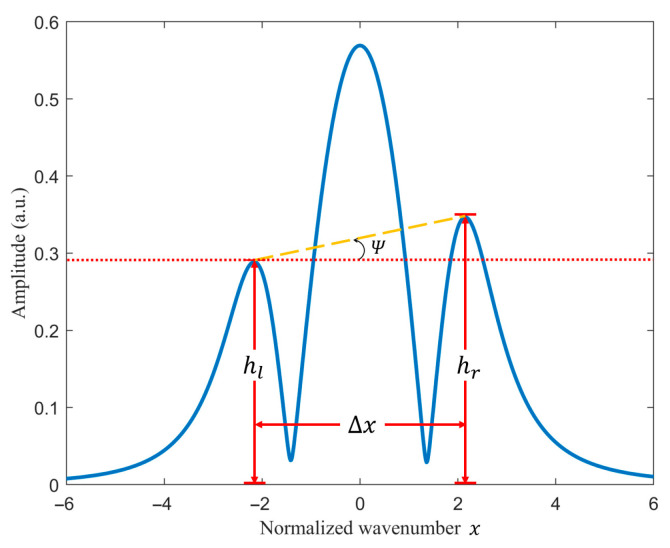
Lateral Peak Inclination Angle on Second harmonic.

The waveform of $X_{2f}$ is illustrated in Figure 1. When x=0, $X_{2f}$ reaches its maximum value, corresponding to the peak of the second-harmonic signal. As indicated by Equations (8) and (20), the presence of amplitude and phase discrepancies between the intensity modulation and frequency modulation introduces additional harmonic components and their cross-terms into the quadrature-demodulated second harmonic. Consequently, the resulting waveform becomes distorted and no longer preserves the symmetry predicted by the theoretical WMS model.

As shown in Figure 1, two local maxima—referred to as lateral peaks—appear on both sides of the principal peak. Let $h_{l}$ and $h_{r}$ denote the amplitudes of the left and right lateral peaks, respectively, and let Δx represent the normalized wave-number difference between these two extrema. The degree of asymmetry induced by intensity-modulation effects can be quantified using the Lateral Peak Inclination Angle (LPIA), denoted as ψ, which is defined as follows:

(21) $$\psi =\arctan\frac{h_{r}-h_{l}}{\Delta x}$$

Evidently, in the conventional theoretical analysis, the second-harmonic component $H_{2}$ exhibits perfect centro-symmetry, yielding ψ=0. However, in the presence of intensity modulation, the quadrature-demodulated signal $X_{2f}$ is jointly influenced by the intensity-modulation amplitude $\delta I_{m}$ and the phase offset ϕ. When ψ>0, the two lateral peaks of the second-harmonic waveform become asymmetric, with the left peak lower than the right; conversely, when ψ<0, the left peak exceeds the right in amplitude. The absolute magnitude $\left|\psi \right|$ characterizes the severity of the distortion in the second-harmonic lineshape.

### 2.3. Harmonic Component Fitting and Decoupling Method Based on Maximum Cross-Correlation Coefficient

To address the waveform asymmetry of the second-harmonic signal induced by residual amplitude modulation, this study proposes a RAM-suppression method based on multiple harmonic component decoupling (MHCD). The first step of the algorithm is to construct a fitting model based on the linear superposition of harmonic components, which is used to estimate the amplitude ratios of the odd-order harmonics embedded in the measured $X_{2f}$ signal. As discussed in the previous section, the numerical value of $X_{2f}$ can be expressed as a sum of products of the first, second, and third harmonics in specific proportions. When the amplitude $\delta I_{m}$ and phase ϕ of the intensity modulation are uniquely defined, these product ratios are uniquely determined. However, it is difficult to measure the modulation amplitude and phase in real time with any practical sensing device. Moreover, due to the long-term instability between the laser driving current and the output optical power, the amplitude and phase of the residual intensity modulation can be regarded as time-varying parameters. Therefore, the proposed fitting approach numerically models the final demodulated $X_{2f}$ signal under uncertain modulation amplitude $\delta I_{m}$ and phase ϕ, enabling the estimation of the amplitude ratios between the odd-order harmonics and the second harmonic from the fitted model.

As indicated by Equation (13), the frequency components of the demodulated signal at twice the modulation frequency of the sinusoidal drive are primarily composed of a linear superposition of the first ($H_{1}$), second ($H_{2}$), and third ($H_{3}$) harmonics. By treating the linear coefficients preceding each harmonic component as undetermined parameters $k_{1}$, $k_{2}$, and $k_{3}$, a linear superposition fitting model—referred to as the harmonic composite operator—can be constructed, which is expressed as follows:

(22) $$\Phi_{2}=k_{1}H_{1}+k_{2}H_{2}+k_{3}H_{3}$$

Here, $k_{1}$, $k_{2}$, and $k_{3}$ correspond to the coefficients associated with the first-, second-, and third-harmonic components, respectively. After performing lock-in demodulation, the resulting ${X^{\prime }}_{2f}$ signal can be expressed as follows:

(23) $$\begin{array}{cc} X_{2f}^{\prime } & =\sqrt{\Phi_{2}^{2}}\\ & =\sqrt{k_{1}^{2}H_{1}^{2}+k_{2}^{2}H_{2}^{2}+k_{3}^{2}H_{3}^{2}+2k_{1}k_{2}H_{1}H_{2}+2k_{2}k_{3}H_{2}H_{3}+2k_{1}k_{3}H_{1}H_{3}} \end{array}$$

To minimize the discrepancy between the reconstructed signal and the actual demodulated signal, the following optimization objective is defined for the aforementioned reconstruction model:

(24) $$\begin{array}{cc} J & =\underset{k_{1},k_{2},k_{3}\in R}{\min}{\left\|X_{2f}-X_{2f}^{\prime }\right\|}^{2}\\ & =\underset{k_{1},k_{2},k_{3}\in R}{\min}{\left\|X_{2f}-\left|k_{1}H_{1}+k_{2}H_{2}+k_{3}H_{3}\right|\right\|}^{2} \end{array}$$

To achieve minimal error between the measured signal and the reconstructed signal, this study employs the waveform cross-correlation coefficient as the optimization criterion for waveform reconstruction. The cross-correlation function between the two signals is expressed as follows in Equation (25):

(25) $$Cov\left(X_{2f},\left|\Phi_{2}\right|\right)=\int_{-\infty }^{\infty }X_{2f}\left(x\right)\cdot \left|\Phi_{2}\left(x+\tau \right)\right|dx$$

Here, τ denotes the frequency offset between the two signals. The harmonic composite operator contains three known harmonic components. When a linear convolution operation is performed, the similarity between the $X_{2f}$ signal and each of the three harmonic components is effectively evaluated. At the frequency corresponding to the maximum of the cross-correlation function, the similarity between $X_{2f}$ and $\Phi_{2}$ is maximized, and the normalized error between the two signals is minimized.

This study is based on the assumption that, for different combinations of $k_{1}$, $k_{2}$, and $k_{3}$, the larger the cross-correlation function between the reconstructed harmonic composite operator and the measured signal, the smaller the error between the actual and reconstructed signals. Among the harmonic composite operators corresponding to a given set of harmonic ratios, the operator with the maximum cross-correlation coefficient achieves the minimum error as defined in Equation (23). Under this condition, the optimization objective can be expressed as follows:

(26) $$J^{\prime }=\underset{j}{\max}\underset{\tau }{\max}\frac{\int_{-\infty }^{\infty }X_{2f}\left(x\right)\cdot \left|\Phi_{2,j}\left(x+\tau \right)\right|dx-E\left(X_{2f}\right)E\left(\left|\Phi_{2,j}\right|\right)}{\sqrt{D\left(X_{2f}\right)}\sqrt{D\left(\left|\Phi_{2,j}\right|\right)}}$$

The objective of the optimization function $J^{\prime }$ is first to determine the optimal cross-correlation coefficient for the harmonic composite operator under the *j*-th set of harmonic ratios, and then to identify the operator with the maximum cross-correlation coefficient overall. Once the optimization function $J^{\prime }$ is achieved, the coefficients $k_{1}$, $k_{2}$, and $k_{3}$ within the harmonic composite operator represent the optimal amplitude ratios of the respective harmonic components in the original $X_{2f}$ signal.

The second step of the MHCD algorithm involves using the harmonic component ratios obtained in the previous step to perform a linear calculation on the harmonic matrix, thereby correcting the waveform of the second-harmonic signal. During the process of determining the optimal harmonic amplitude ratios, a three-dimensional search is required. To simplify the algorithm, both sides of Equation (22) are divided by $k_{2}(k_{2}>0)$, yielding:

(27) $$\frac{\Phi_{2}}{k_{2}}=\frac{k_{1}}{k_{2}}H_{1}+H_{2}+\frac{k_{3}}{k_{2}}H_{3}$$

After performing the aforementioned linear transformation, it can be demonstrated that the new harmonic composite operator yields the same result as the original harmonic composite operator when computing the cross-correlation coefficient:

(28) $$\rho \left(X_{2f},\left|\frac{\Phi_{2}}{k_{2}}\right|\right)=\rho \left(X_{2f},\left|\Phi_{2}\right|\right)$$

Following the linear transformation in Equation (27), the search space for the optimal harmonic amplitude ratios is reduced from three dimensions to two dimensions, let $a_{2}=k_{1}/k_{2},b_{2}=k_{3}/k_{2}$, Equation (27) can be changed as:

(29) $$\Phi_{2}=a_{2}H_{1}+H_{2}+b_{2}H_{3}$$

Under the optimization objective of the function $J^{\prime }$, the absolute value of the optimal harmonic composite operator can be determined. The corresponding optimal harmonic composite operator is then obtained as follows:

(30) $$\Phi_{2}=\mathrm{sgn}\left(\Phi_{2}\right)\cdot \left|\Phi_{2}\right|$$

Here, sgn(x) denotes the sign function. Once the optimal ratios a and b are obtained, the second-harmonic signal $X_{2f}$, along with the first- and third-harmonic signals $X_{1f}$ and $X_{3f}$, are substituted into $\Phi_{2}$, $H_{1}$, and $H_{3}$ in Equation (27), respectively. This yields the second-harmonic component with the odd-order harmonics removed:

(31) $$H_{2}=X_{2f}-a_{2}^{*}X_{1f}-b_{2}^{*}X_{3f}$$

It should also be noted that the first- and third-harmonic signals, $X_{1f}$ and $X_{3f}$, contain even-order harmonic components. Consequently, directly subtracting $X_{1f}$ and $X_{3f}$ from $X_{2f}$ would introduce additional coupled harmonic components at other frequencies. To achieve full-band harmonic decoupling, waveform reconstruction and compensation must also be applied to the first- and third-harmonic signals. Accordingly, the first-harmonic composite operator $\Phi_{1}$ and the third-harmonic composite operator $\Phi_{3}$ are formulated as follows:

(32) $$\Phi_{1}=a_{1}H_{1}+b_{1}H_{2}$$

(33) $$\Phi_{3}=a_{3}H_{2}+H_{3}$$

where a∈(−1,1), $b_{1}$, $a_{3}\in R$. Due to the presence of DC offsets, the optimization objective for the first-harmonic composite operator $\Phi_{1}$ is to ensure that $\Phi_{1}$ matches $X_{1f}$ as closely as possible. Similarly, the optimization objective for the third-harmonic composite operator $\Phi_{3}$ is to ensure that $\left|\Phi_{3}\right|$ closely approximates $X_{3f}$. In Equations (32) and (33), neither the zero-order nor the fourth-order harmonic components are included; instead, the fitting is performed solely using their own harmonic component and the second harmonic. After correcting both the first- and third-harmonic signals, the decoupling of multiple harmonic components at the 1*f*, 2*f*, and 3*f* frequencies is achieved using the following formulation:

(34) $$\left[\begin{array}{ccc} a_{1}^{*} & b_{1} & 0\\ a_{2}^{*} & 1 & b_{2}^{*}\\ 0 & a_{3}^{*} & 1 \end{array}\right]\left[\begin{array}{c} H_{1}\\ H_{2}\\ H_{3} \end{array}\right]=\left[\begin{array}{c} X_{1f}\\ X_{2f}\cdot \mathrm{sgn}\left(\Phi_{2}\right)\\ X_{3f}\cdot \mathrm{sgn}\left(\Phi_{3}\right) \end{array}\right]$$

## 3. Simulation Analysis

Based on the theoretical analysis described above, this study establishes a mathematical model of the MHCD algorithm in MATLAB (R2021a) and conducts principle-level simulations. This section first examines the influence of optical-intensity modulation terms—namely the modulation amplitude and modulation phase—on the waveform characteristics of different harmonic orders, and subsequently uses these observations to verify the theoretical feasibility of the proposed algorithm.

### 3.1. Influence of Optical-Intensity Modulation Parameters on Harmonic Characteristics

**Figure 2:**
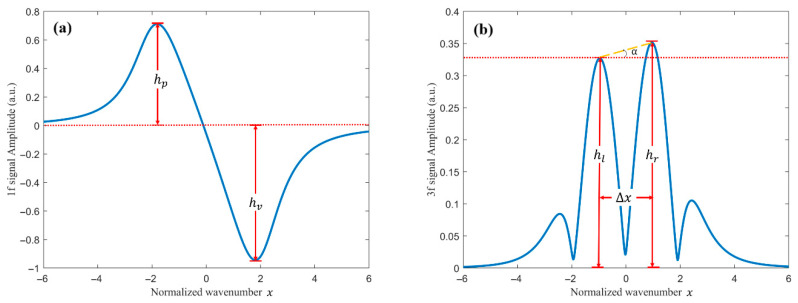
(**a**) Peak–valley ratio of the first-harmonic signal; (**b**) double-peak inclination angle of the third-harmonic signal.

In this work, the Lateral Peak Inclination Angle (LPIA) is employed as the criterion for evaluating the asymmetry of the second-harmonic signal. Under the influence of residual amplitude modulation, the first- and third-harmonic signals also exhibit waveform asymmetry [38]. The asymmetry of the first harmonic is primarily manifested as unequal peak and valley magnitudes. Accordingly, the peak–valley ratio (PVR) is defined as the metric for assessing its waveform symmetry, as illustrated in Figure 2a. For the third harmonic, the asymmetry is mainly reflected in the unequal amplitudes of the left and right peaks. Therefore, the double-peak inclination angle (DPIA) is introduced as the metric for characterizing its waveform symmetry, as shown in Figure 2b.

#### 3.1.1. Effect of the Intensity Modulation Phase

**Figure 3:**
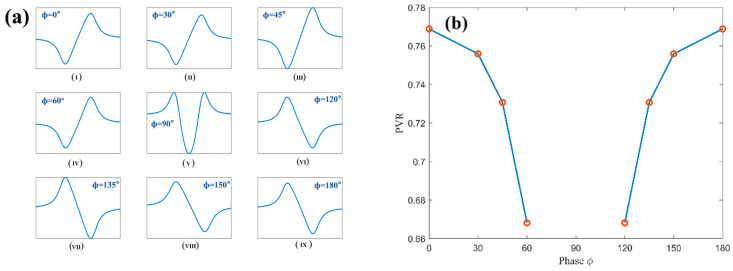
(**a**) The evolution of the first-harmonic waveform as the phase varies from (**I**–**IX**) 0° to 180°; (**b**) the corresponding variation in the first-harmonic peak–valley ratio (PVR).

With the intensity modulation amplitude fixed at $\delta I_{m}=2$, the phase ϕ is varied over the range of 0~180°. The quadrature demodulated signals at the fundamental frequency and at the second and third harmonics of the sinusoidal modulation signal are then obtained, respectively. Figure 3 illustrates the variation in the first-harmonic waveform and the corresponding peak–valley ratio as the phase changes from 0~180°.

**Figure 4:**
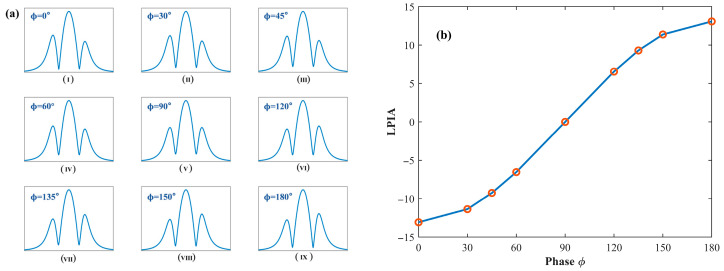
(**a**) The evolution of the second-harmonic waveform as the phase varies from (**I**–**IX**) 0° to 180°; (**b**) the corresponding variation in the lateral peak inclination angle (LPIA).

From the two figures above, it can be observed that when the phase ϕ is equal to 90°, the waveform loses the characteristic features of the first harmonic and can no longer be used as a feature signal for concentration retrieval; therefore, this case is not further discussed. When $\phi <90^{\circ }$, the valley of the first-harmonic waveform appears on the left side of x=0, while the peak appears on the right side of x=0. In this case, the peak value is consistently smaller than the valley value, and as ϕ approaches 90°, the difference between the peak and valley increases, resulting in a smaller PVR. When $\phi >90^{\circ }$, the waveform polarity is reversed: the peak appears on the left side of x=0, whereas the valley appears on the right side. Similarly, as ϕ approaches 90°, the PVR decreases. These results indicate that the intensity modulation phase not only affects the symmetry of the first-harmonic signal but also causes a polarity inversion, which explains why the first-harmonic term in Equation (32) can take either positive or negative values. Figure 4 shows the evolution of the second-harmonic waveform and Lateral Peak Inclination Angle (LPIA) as the phase varies in the range of 0~180°.

**Figure 5:**
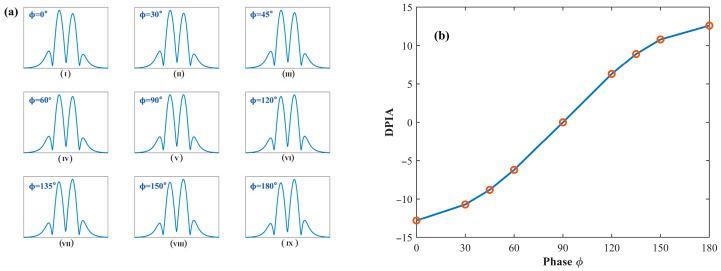
(**a**) The evolution of the third-harmonic waveform as the phase varies in the range of (**I**–**IX**) 0° to 180°; (**b**) the corresponding variation in the double-peak inclination angle (DPIA).

As shown in the figure, the maximum peak value of the quadrature-demodulated signal in $X_{2f}$ is essentially insensitive to variations in the intensity modulation phase, and its waveform corresponds to the absolute value of the theoretical model $H_{2}$. When $\phi =90^{\circ }$, the lateral peak inclination angle (LPIA) is zero, indicating a fully symmetric line shape. When $\phi <90^{\circ }$, the LPIA is negative, characterized by a higher left-side peak than the right-side peak. Conversely, when $\phi >90^{\circ }$, the LPIA becomes positive, with the left-side peak lower than the right-side peak. Moreover, as the phase gradually approaches 90°, the LPIA converges toward zero, and the waveform symmetry is progressively improved. Figure 5 shows the evolution of the third-harmonic waveform and the corresponding double peak inclination angle as the phase varies from 0~180°.

As shown in the figure, under the influence of residual amplitude modulation, the quadrature-demodulated signal $X_{3f}$ exhibits a zero baseline, and its waveform corresponds to the absolute value of the theoretical model $H_{3}$. When the phase $\phi =90^{\circ }$, the DPIA equals zero, indicating a perfectly symmetric line shape. When $\phi <90^{\circ }$, the left peak is higher than the right peak, resulting in a negative DPIA. Conversely, when $\phi >90^{\circ }$, the left peak becomes lower than the right peak, leading to a positive DPIA. Moreover, as the phase gradually approaches 90°, the DPIA approaches zero, and the waveform symmetry is progressively improved.

#### 3.1.2. Effect of the Intensity Modulation Amplitude

**Figure 6:**
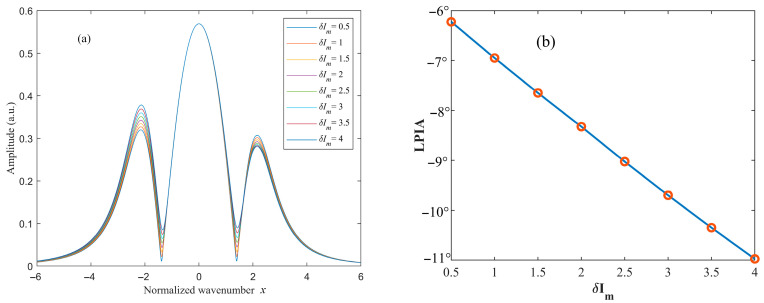
Variation in the (**a**) 2*f* signal and the (**b**) LPIA with respect to the intensity modulation amplitude ($\phi =30^{\circ }$).

**Figure 7:**
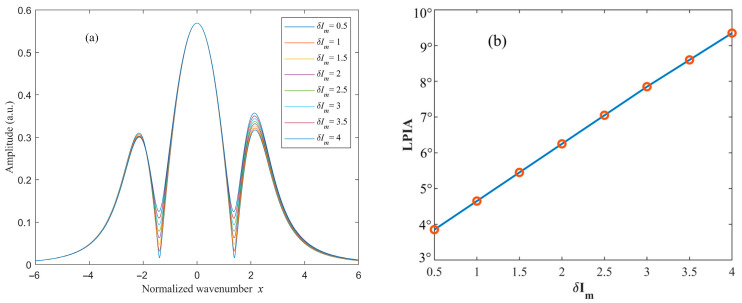
Variation in the (**a**) 2*f* signal and (**b**) the LPIA with respect to the intensity modulation amplitude ($\phi =120^{\circ }$).

With the phase ϕ fixed, the amplitude of residual amplitude modulation $\delta I_{m}$ is varied, and the simulated results are shown in Figure 6 and Figure 7. The quadrature-demodulated signals at twice the modulation frequency under different amplitude modulation depths $\delta I_{m}$, as functions of the normalized wavenumber x, are illustrated in Figure 6a and Figure 7a. When $\phi =30^{\circ }$, the LPIA decreases with increasing $\delta I_{m}$, indicating a degradation in waveform symmetry. In contrast, when $\phi =120^{\circ }$, the LPIA increases as $\delta I_{m}$ increases. These results demonstrate that variations in $\delta I_{m}$ do not alter the polarity of the LPIA but exacerbate the degree of waveform asymmetry.

From the above discussion, it can be concluded that the fundamental origin of waveform asymmetry lies in the linear component of the intensity modulation, specifically, the existence of a certain phase difference ϕ between the intensity modulation and the frequency modulation. Taking 90° as the reference, when the phase is below or above 90°, the polarity of the lateral peak inclination angle (LPIA) of the second harmonic is reversed. An increase in the intensity modulation amplitude $\delta I_{m}$ leads to a larger absolute value of the LPIA, thereby exacerbating the waveform asymmetry. Since the baselines of the second- and third-harmonic signals are zero, their negative portions are converted to positive values after quadrature demodulation. In contrast, the baseline of the first-harmonic signal is significantly larger than its harmonic amplitude, so the quadrature-demodulated first-harmonic signal represents an inversion of the original signal. Based on the relationship between the phase-locked demodulated signals and the original signals, the reconstructed waveforms of the second and third harmonics need to be multiplied by a sign function to recover the original signal, whereas the first-harmonic reconstructed waveform inherently possesses both positive and negative polarity. In the first-harmonic composite operator in Equation (31), $a_{1}$ is assigned values of −1 and 1 to match the polarity of the demodulated signal.

### 3.2. Simulation of the MHCD Algorithm

**Figure 8:**
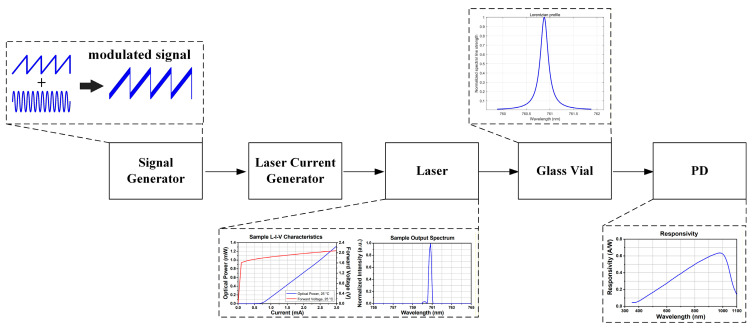
The generation of the laser drive modulation signal and the acquisition process of the absorption spectrum.

**Figure 9:**
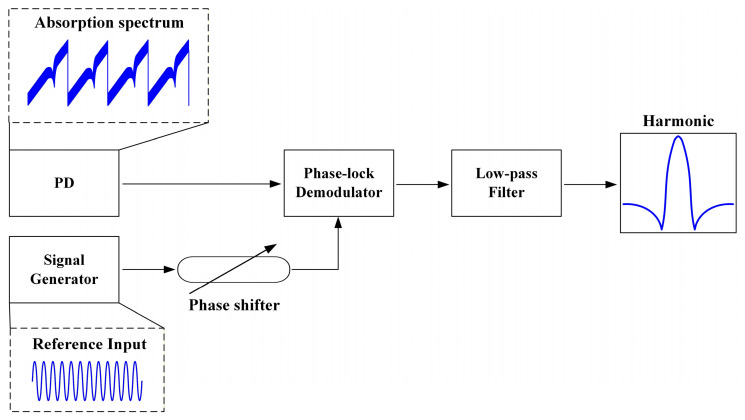
Extracting harmonic signals by phase-lock demodulation.

To verify the compensation capability of the multiple harmonic component decoupling algorithm for different types of harmonic asymmetry, a mathematical model of the residual oxygen detection system for sealed pharmaceutical vials was established. Figure 8 illustrates the generation of the laser drive modulation signal and the acquisition process of the absorption spectrum signal, while Figure 9 depicts the procedure for extracting harmonic signals using phase-lock demodulation.

According to the HITRAN database, an isolated absorption line of O_2_ at 760.885 nm (13,142.6 cm^−1^) was selected as the target absorption line. The frequency of the sawtooth scanning signal was set to 50 Hz with a scanning amplitude of 1.4 mA, while the sinusoidal modulation signal had a frequency of 15 kHz and an amplitude of 0.12 mA. The sampling frequency was set to 5 MHz. The ambient temperature was set to 296 K and the pressure was assumed to be 1 atm, under which the absorption line strength of O_2_ at 13,142.6 cm^−1^ is 8.847 cm^−1^/(molecule·cm^−2^). The phase ϕ of the intensity modulation was varied from 0° to 360°, and the amplitude $\delta I_{m}$ of the intensity modulation was varied from 0.5 to 4, which essentially covers the full range of experimental conditions. To better reflect practical conditions in the simulation, the oxygen concentration inside the vial was set to 0%, whereas the oxygen concentration outside the vial was set to 21%. The effective absorption path length inside the vial was 22 mm, and that outside the vial was 80 mm.

**Figure 10:**
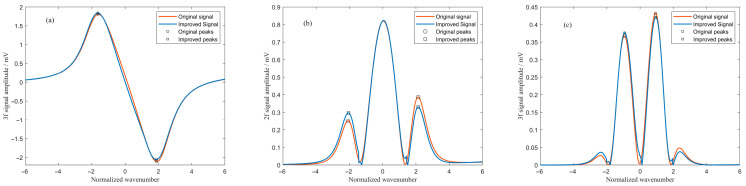
Performance of the MHCD algorithm in optimizing the (**a**) first-, (**b**) second-, and (**c**) third-harmonic signals under a representative operating condition.

The experimental results demonstrate that the MHCD algorithm achieves satisfactory optimization across the specified range of intensity modulation parameters. It not only provides accurate fitting for demodulated signals across different frequency components but also effectively restores the waveform symmetry of each harmonic component. Figure 10 illustrates the algorithm’s optimization performance for the demodulated signals at a typical operating point (intensity modulation phase 180°, amplitude 2). Specifically, for the first-harmonic signal, the original characteristic of the peak being lower than the valley was corrected: after processing, the peak was enhanced while the valley was correspondingly reduced, resulting in a more symmetric waveform. In the processing of the second-harmonic signal, the algorithm effectively eliminated the pronounced lateral peak inclination in the original signal, bringing the inclination angle close to the ideal zero value. For the third-harmonic signal, the algorithm successfully corrected the asymmetry of the original waveform, where the right peak was higher than the left, thereby reducing the amplitude difference between the two peaks.

**Figure 11:**
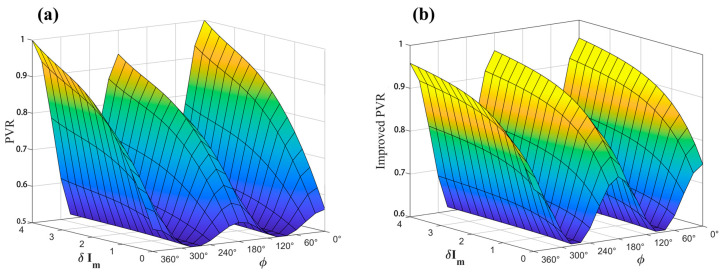
PVR comparison before and after MHCD algorithm optimization. (**a**) Original PVR; (**b**) Improved PVR.

**Figure 12:**
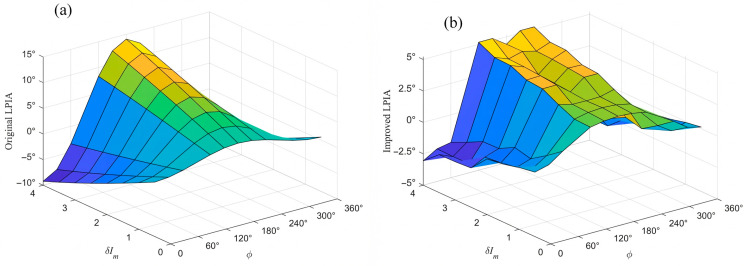
LPIA comparison before and after MHCD algorithm optimization. (**a**) Original LPIA; (**b**) Improved LPIA.

**Figure 13:**
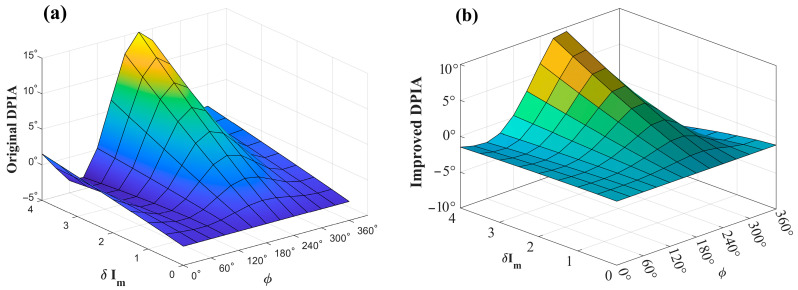
DPIA comparison before and after MHCD algorithm optimization. (**a**) Original DPIA; (**b**) Improved DPIA.

The variations in the geometric asymmetry characteristics of the first-, second-, and third-order harmonics before and after application of the multi-harmonic component decoupling (MHCD) algorithm—quantified by PVR, LPIA, and DPIA, respectively—are presented in Figure 11, Figure 12 and Figure 13. For clarity, the principal observations are summarized below.

(1) First harmonic (PVR):

Prior to algorithmic processing, the peak-to-valley ratio ranged from [0.52, 0.98]. After application of the MHCD algorithm, this range shifted to [0.64, 0.96]. This change suggests that the algorithm tends to moderate the amplitude imbalance of the first harmonic, with peak and valley magnitudes exhibiting a comparatively more balanced distribution.

(2) Second harmonic (LPIA):

In the absence of processing, the side-lobe inclination angle of the second harmonic spanned [−9.2°, 14.4°], with extrema occurring at 180°, consistent with the theoretical analysis discussed previously. Following MHCD processing, the inclination angle range of the dual valleys was reduced to [−2.8°, 5°]. This contraction indicates that the geometric asymmetry of the second-harmonic waveform is alleviated to a certain extent.

(3) Third harmonic (DPIA):

For the unprocessed signal, the dual-peak inclination angle was in the range of [−3.9°, 15°]. After implementation of the MHCD algorithm, this interval decreased to [−2.6°, 9.7°], indicating a certain degree of reduction in asymmetry for the third harmonic.

**Figure 14:**
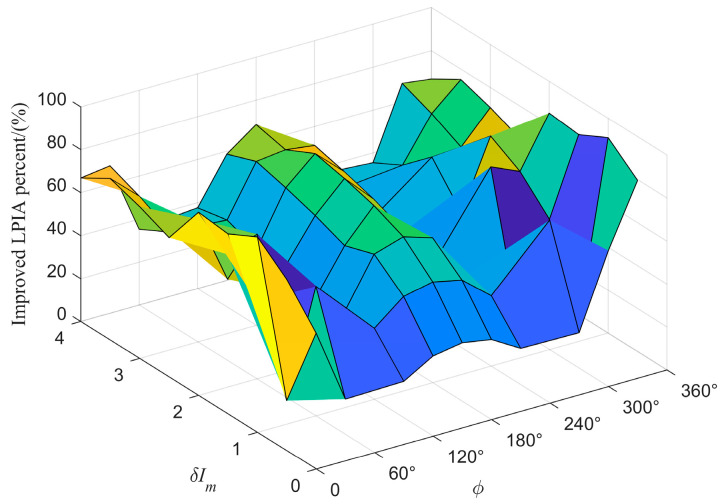
LPIA improvement percentage.

Considering that the second harmonic is commonly employed for concentration retrieval in TDLAS-WMS systems, a further quantitative analysis is conducted to assess the effectiveness of MHCD in improving LPIA, which is defined as follows.

(35) $$\Delta =\frac{\psi_{pre}-\psi_{post}}{\psi_{pre}}\times 100\%$$

where $\psi_{pre}$ denotes the original LPIA, and $\psi_{post}$ denotes the LPIA after algorithmic improvement. The LPIA improvement percentage is shown in Figure 14.

By combining Figure 12 and Figure 14, it can be observed that the optimization performance of the algorithm is positively correlated with the initial degree of harmonic asymmetry. When the initial peak–valley angle is large (i.e., the harmonic asymmetry is pronounced), the algorithm exhibits a significant optimization effect, with a maximum improvement percentage of 70%. Conversely, when the initial peak–valley angle is small, the magnitude of signal adjustment is relatively limited, indicating that the MHCD algorithm is adaptive and capable of dynamically adjusting the optimization strength according to the initial degree of harmonic asymmetry, thereby avoiding over-processing. This characteristic enhances its general applicability and reliability in practical applications.

### 3.3. Accuracy Assessment of Fitting

This section aims to verify that the proposed algorithm maintains high fitting accuracy under the conditions described in Section 3.1. The simulation was conducted with the phase varying over the range $\phi \in [0^{\circ },360^{\circ }]$ and the amplitude over the range $\delta I_{m}\in [0.5,4]$, in order to cover as many experimental scenarios as possible. Based on these parameters, quadrature-demodulated signals at the fundamental, second, and third harmonics (1*f*, 2*f*, 3*f*) were generated, with a total of N=800 sampling points. Subsequently, the harmonic composite operators for the first, second, and third harmonics were constructed, and their cross-correlation coefficients were calculated under different parameter sets. The harmonic composite operator exhibiting the maximum cross-correlation coefficient was selected as the optimal fitted value. Since multiplying the harmonic composite operator by different linear coefficients yields equivalent results, both the demodulated signals and the harmonic composite operators were normalized to facilitate comparison of the model error between them. The normalized root-mean-square error (RMSE) was then employed as the metric for evaluating the fitting performance of the model.

The specific implementation steps are as follows:

Step1: Based on Equations (28), (30) and (31), the composite operators for the first, second, and third harmonics were generated. The amplitude ratio ranges for the first-harmonic composite operator were set as $a_{1}\in [-1,1]$ and b∈[−0.2,0.2]; for the second-harmonic composite operator, $a_{2}\in [-0.2,0.2]$ and $b_{2}\in [-0.2,0.2]$; and for the third-harmonic operator, $a_{3}\in [-0.2,0.2]$. The search step size for the harmonic composite operators was adjustable, and in this paper, it was set to 0.01.

Step2: The reconstructed signals $\Phi_{1,j}$, $\left|\Phi_{2,j}\right|$, and $\left|\Phi_{3,j}\right|$ were cross-correlated with the demodulated signals $X_{1f}$, $X_{2f}$, and $X_{3f}$, respectively. The position corresponding to the maximum cross-correlation coefficient was identified as the optimal location for the *j*-th amplitude ratio combination.

Step3: The harmonic composite operator exhibiting the maximum cross-correlation coefficient among the set of j amplitude ratio combinations was selected as the optimal composite operator $\left(\Phi_{1,j},\left|\Phi_{2,j}\right|,\left|\Phi_{3,j}\right|\right)$. The corresponding harmonic amplitude ratios $\left(a_{1}^{*},b_{1}^{*},a_{2}^{*},b_{2}^{*},a_{3}^{*}\right)$ represent the output of the first step of the MHCD algorithm and serve as inputs for the subsequent linear matrix operations.

**Figure 15:**
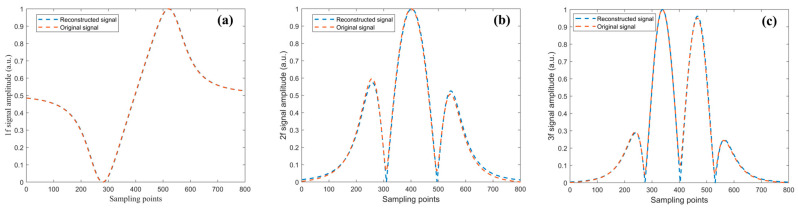
(**a**) 1*f*, (**b**) 2*f*, (**c**) 3*f* signals with the optimal harmonic composite operator.

The optimal harmonic composite operator obtained in Step S3, along with the fitted curves for each harmonic, is shown in Figure 15. The smaller the error between the reconstructed signals and the original signals, the higher the accuracy of the harmonic amplitude ratios obtained through inversion.

To further quantitatively validate the fitting accuracy of the optimal harmonic composite operator, simulation data were generated by sampling $\delta I_{m}\in [0.5,4]$ at intervals of 0.5 and $\phi \in [0^{\circ },360^{\circ }]$ at intervals of 30°, yielding a total of 88 sets of 1*f*, 2*f*, and 3*f* signals. Each set was cross-correlated with its corresponding harmonic composite operator, and the optimal harmonic composite operator was selected to reconstruct the signals.

The original demodulated signals were treated as the ground truth and, together with the reconstructed signals, were normalized to the range [0,1]. The root-mean-square error (RMSE) was employed to evaluate the model fitting performance, which is defined as follows:

(36) $$RMSE=\sqrt{\frac{1}{N}\sum_{i=1}^{N}{\left(X_{nf}^{\prime }\left(i\right)-\Phi_{n}^{\prime }\left(i\right)\right)}^{2}}$$

Here, ${X^{\prime }}_{nf}$ denotes the normalized value of the n-th harmonic demodulated signal, and ${\Phi^{\prime }}_{n}$ represents the normalized value of the corresponding optimal harmonic composite operator.

The average RMSE values of the 88 sets of original and reconstructed signals are summarized in Table 1. The proposed algorithm exhibits high fitting accuracy under all 88 intensity modulation parameter conditions, with the average RMSE for each harmonic component being lower than 0.01. These results demonstrate that the algorithm has strong adaptability across diverse application scenarios and is capable of accurately fitting harmonic components over the entire frequency range.

### 3.4. Noise Robustness Analysis

**Figure 16:**
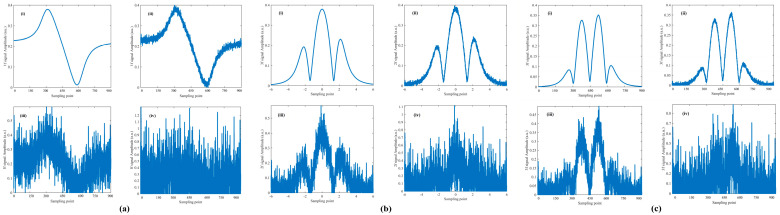
(**a**) First, (**b**) Second, (**c**) Third harmonic in Gaussian noise with different signal-to-noise ratios: (**i**) no noise, (**ii**) SNR = 28.5 dB, (**iii**) SNR = 8.5 dB, (**iv**) SNR = −2.5 dB.

**Figure 17:**
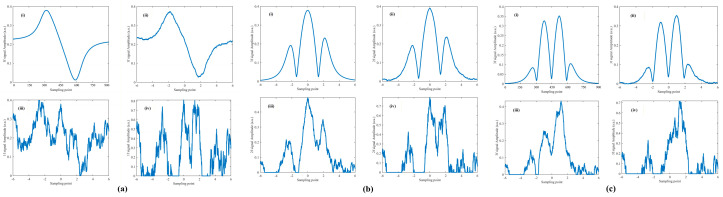
(**a**) First, (**b**) Second, (**c**) Third harmonic in baseline drift with different signal-to-noise ratios: (**i**) no noise, (**ii**) SNR = 28.5 dB, (**iii**) SNR = 8.5 dB, (**iv**) SNR = −2.5 dB.

In gas detection applications under open environments, the system is inevitably contaminated by environmental noise sources such as strong electromagnetic interference, temperature and humidity fluctuations, and external light disturbances. Therefore, the proposed MHCD algorithm must exhibit sufficient noise immunity to accurately recover the original harmonic amplitude ratios from noisy signals. Since Gaussian noise shares statistical characteristics similar to those commonly encountered in TDLAS systems, and baseline drift noise can effectively simulate the impact of environmental parameter drift on the hardware of the detection system, Gaussian noise and baseline drift noise with different intensities were superimposed onto the noise-free demodulated signals to evaluate the noise robustness of the MHCD algorithm. Four noise conditions were considered, namely noise-free, low-level noise, medium-level noise, and high-level noise, corresponding to signal-to-noise ratios (SNRs) of noise-free, 28.5 dB, 8.5 dB, and −2.5 dB, respectively. The demodulated signals contaminated by different noise levels are illustrated in Figure 16 and Figure 17.

**Figure 18:**
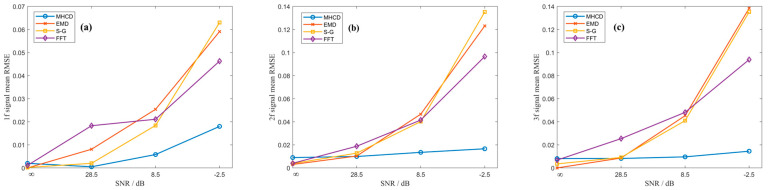
Performance comparison of the MHCD algorithm under Gaussian noise and three reference methods in reconstructing the original signals using the (**a**) first-, (**b**) second-, and (**c**) third-harmonic components.

**Figure 19:**
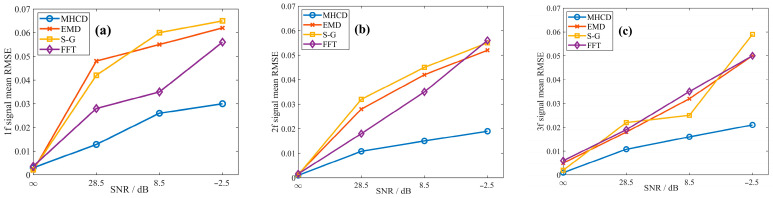
Performance comparison of the MHCD algorithm under baseline drift and three reference methods in reconstructing the original signals using the (**a**) first-, (**b**) second-, and (**c**) third-harmonic components.

In addition, three commonly used waveform reconstruction methods—empirical mode decomposition (EMD), the Savitzky–Golay (S-G) filter, and the fast Fourier transform (FFT)—were selected for comparative evaluation under the same test conditions. The reconstructed signals obtained using these methods were subjected to the same normalization procedure, and the average RMSE was used to quantitatively assess their performance. The average RMSE values achieved by each algorithm in fitting the demodulated signals are presented in Figure 18 and Figure 19 and Table 2. The MHCD algorithm exhibits comparable average RMSE to the EMD, S-G, and FFT methods under noise-free conditions, while achieving the lowest RMSE under low-, medium-, and high-noise conditions. This performance advantage arises because the construction of the harmonic composite operators incorporates substantial prior information, including the modulation index and spectral linewidth, enabling the algorithm to effectively recover clean harmonic signals even in the presence of noise.

By jointly considering the results presented in Section 3.2 and Section 3.3, it can be concluded that the proposed method maintains high fitting accuracy over a wide range of intensity modulation parameter variations, demonstrating strong adaptability to measurement scenarios in which the intensity modulation parameters are uncertain and randomly varying. Moreover, in comparative experiments involving demodulated signals corrupted by different noise levels, the performance of the proposed method remains essentially stable as the signal-to-noise ratio decreases, exhibiting superior noise robustness compared with conventional algorithms. Consequently, the proposed approach is capable of effectively suppressing the influence of residual amplitude modulation on the waveforms of multiple harmonic components.

## 4. Experiments

### 4.1. Experimental System

**Figure 20:**
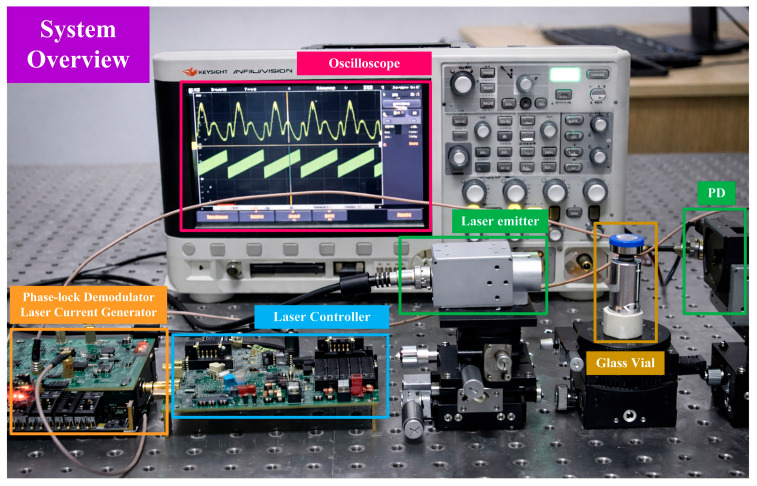
The experimental setup for residual oxygen detection in sealed pharmaceutical vials.

The experimental system was composed of a ZYNQ-7020 development board (AX7021 V2.0, ALINX, Shanghai, China) and a custom-designed hardware board, which together were used to implement laser drive signal generation, absorption spectrum acquisition, and data transmission to the host computer, as illustrated in Figure 20.

The ZYNQ-7020 platform generated modulation signals based on direct digital synthesis (DDS). The ramp scanning signal had a frequency of 50 Hz with a scan amplitude of 1.4 mA, while the sinusoidal modulation signal operated at a frequency of 15 kHz with an amplitude of 0.12 mA. These digital signals were transmitted to the custom hardware board, where digital-to-analog conversion and signal conditioning were performed to generate analog current modulation signals suitable for driving the semiconductor laser (L760VH1, Thorlabs, Newton, NJ, USA). The distance between the laser source and the photodetector was approximately 80 mm. After gas absorption, the transmitted optical signal was incident on a photodetector (PDA36A2, Thorlabs, Newton, NJ, USA), which converted the optical signal into an electrical signal. The resulting absorption spectrum signal was then fed into the same custom hardware board for signal conditioning and analog-to-digital conversion at a sampling rate of 5 MHz, and subsequently transmitted to the host computer via a serial interface for data storage. Furthermore, a commercial laser controller (VITC002, Thorlabs, Newton, NJ, USA) was integrated into the system to control the laser operating temperature and current threshold, ensuring that the laser operated within a safe and stable regime. Collectively, these modules formed a complete system for residual oxygen detection in sealed pharmaceutical vials.

### 4.2. Experiments and Results Analysis

**Figure 21:**
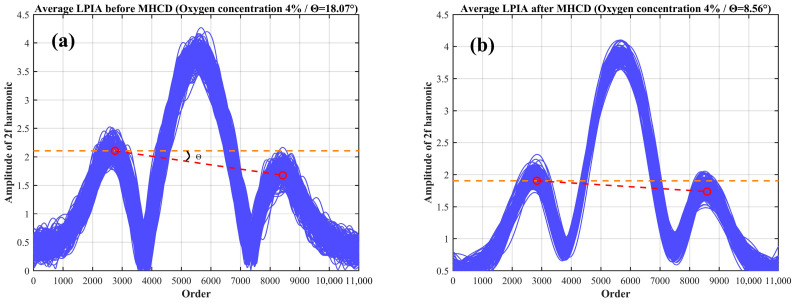
Second-harmonic signals (**a**) before and (**b**) after applying the MHCD algorithm.

To verify the suppression effect of the MHCD algorithm on residual amplitude modulation noise during the detection process, repeatability experiments were conducted using a vial with a diameter of 22 mm and an oxygen concentration of 4% as the test object. A total of 200 measurements were performed. Figure 21a shows the original second-harmonic signal acquired during the experiments. By extracting the normalized coordinates of the peak-to-peak points on the left and right sides of the harmonic signal and connecting their averaged values, the mean LPIA was calculated to be 18.07°, indicating the presence of pronounced RAM noise in the detection process and a significant degradation of the harmonic geometric symmetry. Figure 21b presents the second-harmonic signal after processing with the multi-harmonic component decoupling (MHCD) algorithm. As can be observed, the harmonic waveform exhibits markedly improved symmetry and overall quality after algorithmic processing, while the mean LPIA is reduced to 8.56°. These results clearly demonstrate the effectiveness of the MHCD algorithm in suppressing RAM noise and enhancing the geometric symmetry of harmonic signals.

**Figure 22:**
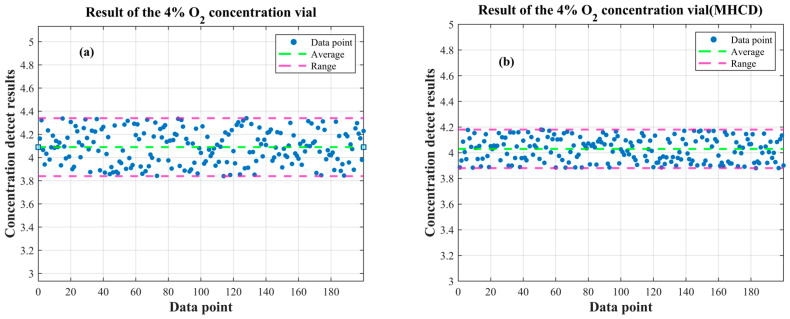
Result of the 4% O_2_ concentration vial (**a**) before and (**b**) after applying the MHCD algorithm.

To further elucidate whether the proposed MHCD algorithm exerts a direct impact on concentration estimation rather than merely improving intermediate waveform characteristics, an additional comparison of the retrieved concentration results was conducted based on the repeatability experiment. Since concentration inversion in TDLAS–WMS is directly derived from the demodulated harmonic signals, any stabilization of the harmonic waveform is expected to propagate to the concentration-estimation stage. Therefore, concentration inversion was performed using the second-harmonic signal shown in Figure 21, and the corresponding concentration retrieval results are presented in Figure 22.

As shown in Figure 22, after applying the MHCD algorithm, the dispersion of the retrieved concentration is significantly reduced. Specifically, the standard deviation decreases from 0.23 to 0.12, and the range decreases from 0.47 to 0.29 over 200 consecutive measurements under identical conditions. This reduction in variability indicates a clear improvement in the repeatability precision of the concentration estimation. The result confirms that the proposed MHCD algorithm produces a direct effect at the concentration-estimation level, rather than merely improving the geometric characteristics of the harmonic waveform.

In the pharmaceutical industry, sterile products packaged in vials are subjected to container closure integrity testing based on a predefined oxygen concentration threshold, according to which the products are inspected, classified, and rejected. The detection performance is typically evaluated using the true positive rate and false positive rate. To further assess the discrimination capability of the MHCD algorithm for vials with low oxygen content and small concentration differences under practical inspection conditions, seven groups of vials with a diameter of 22 mm and an oxygen concentration gradient of 2% were selected as test samples. The corresponding internal oxygen concentrations were 0%, 2%, 4%, 6%, 8%, 10%, and 12%, respectively, and concentration discrimination experiments were conducted.

To quantitatively evaluate the classification performance of multi-concentration samples, this study constructs an interval division method based on decision thresholds between adjacent classes. Let the number of samples in each class be equal, denoted as *M*. The True Positive Rate (TPR) for the *k*-th class is defined as the proportion of samples falling within the corresponding decision interval. The False Negative Rate (FNR) and False Positive Rate (FPR) are defined as the proportions of samples crossing into the adjacent lower concentration and higher concentration intervals, respectively. This definition provides an intuitive representation of the distribution shift between adjacent categories, making it more suitable for threshold-based sequential decision problems.

Suppose there are *N* sample sets arranged in ascending order of concentration, and the number of samples in each category is the same, which is *M*. Then there is:

(37) $$X_{k}=\{x_{k,1},x_{k,2},\cdots ,x_{k,M}\},\;k=1,2,\ldots ,N$$

The discrimination threshold between adjacent categories is defined as:

(38) $$T_{k}=\frac{\underset{1\leq i\leq M}{\max}(x_{k,i})+\underset{1\leq j\leq M}{\min}(x_{k+1,j})}{2},\;k=1,\ldots ,N-1$$

Based on the threshold sequence $\left\{T_{k}\right\}$, the discrimination interval corresponding to the *k*-th class is

(39) $$I_{k}=\left\{\begin{array}{ll} (-\infty ,T_{1}), & k=1\\ {}[T_{k-1},T_{k}), & 2\leq k\leq N-1\\ {}[T_{N-1},+\infty ), & k=N \end{array}\right.$$

Based on the above definition, the TPR of the *k*-th class samples can be defined as the proportion of samples in the *k*-th class that fall within their corresponding discrimination interval $I_{k}$, that is:

(40) $${\mathrm{TPR}}_{k}=\frac{1}{M}\left|\left\{x\in X_{k}|x\in I_{k}\right\}\right|$$

The FPR can be defined as the proportion of the *k*-th type of samples that fall into the upper adjacent interval (in the high concentration direction), that is:

(41) $${\mathrm{FPR}}_{k}=\left\{\begin{array}{ll} \frac{1}{M}\left|\left\{x\in X_{k}|x\geq T_{k}\right\}\right|, & k\leq N-1\\ 0, & k=N \end{array}\right.$$

The FNR can be defined as the proportion of the *k*-th class samples that fall into the lower adjacent interval (in the low concentration direction), that is:

(42) $${\mathrm{FNR}}_{k}=\left\{\begin{array}{ll} 0, & k=1\\ \frac{1}{M}\left|\left\{x\in X_{k}|x<T_{k-1}\right\}\right|, & k\geq 2 \end{array}\right.$$

And for the intermediate category where 2 ≤ *k* ≤ *N* − 1, there is:

(43) $${\mathrm{TPR}}_{k}+{\mathrm{FNR}}_{k}+{\mathrm{FPR}}_{k}=1,\;2\leq k\leq N-1$$

**Figure 23:**
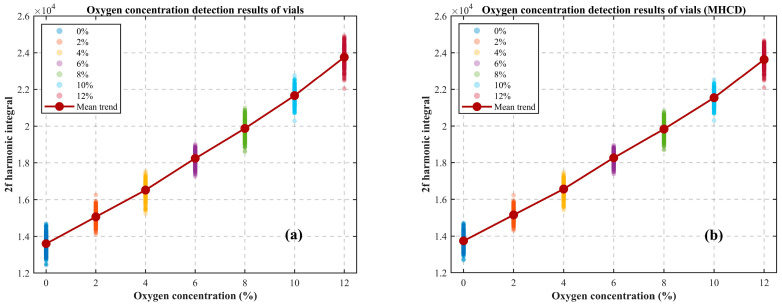
Detection results of vials with a 2% oxygen concentration gradient (**a**) before and (**b**) after applying the MHCD algorithm.

A total of 200 repeated measurements were performed. The acquired absorption spectra were processed using quadrature demodulation to extract the second-harmonic component. An experimental group (with MHCD algorithm processing) and a control group (without algorithm processing) were established for comparison. The integral value of the second-harmonic signal was used as the characteristic parameter for oxygen concentration inversion and classification. The detection results before and after applying the MHCD algorithm are presented as scatter plots in Figure 23a and Figure 23b, respectively, while the corresponding comparisons of true positive rates (TPRs) and false positive rates (FPRs) are summarized in Table 3.

In Figure 23, the horizontal axis represents the oxygen concentration within the vials under test, and the vertical axis corresponds to the integrated value of the second harmonic obtained after quadrature demodulation. Combining the experimental results from Figure 23 and Table 3, it can be seen that, without compromising the overall linearity of the detection results, the MHCD algorithm can significantly enhance the discrimination between vials with low oxygen content and small concentration differences. Specifically, after applying the MHCD algorithm, the system’s TPR increased by 6–11%, while the FPR and FNR were effectively suppressed, with a reduction range of 4–9% and 0.5–7%. These findings indicate that the algorithm improves both the sensitivity and reliability of detecting vials with low oxygen concentration and minimal concentration differences, demonstrating its potential for implementation in practical pharmaceutical industrial online inspection lines.

## 5. Conclusions

This study addresses the influence of residual amplitude modulation noise in TDLAS-WMS systems and proposes a multi-harmonic component decoupling (MHCD) method for RAM noise suppression, aiming to mitigate the degradation in detection performance caused by harmonic asymmetry. First, the distortion mechanisms induced by RAM noise on different harmonic components are systematically analyzed. It is shown that the phase of intensity modulation alters both the magnitude and direction of the side-peak tilt in the second harmonic, while the modulation depth further exacerbates this asymmetry. In addition, the baseline symmetry of the first harmonic and the central-frequency symmetry of the third harmonic are also significantly affected. Based on these observations, the proposed method constructs pure harmonic composite operators and performs cross-correlation operations among harmonics obtained after quadrature demodulation. The optimal harmonic component ratios are determined at the composite operator corresponding to the maximum correlation coefficient, thereby eliminating out-of-band harmonic interference and enabling the accurate reconstruction of a pure second-harmonic signal. The fitting accuracy and noise robustness of the algorithm are subsequently verified.

Finally, a packaged vial residual oxygen detection system is established to experimentally validate the effectiveness of the MHCD algorithm in suppressing RAM noise and improving the detection performance for low-oxygen vials. Repetitive experiments are first conducted using vials with a diameter of 22 mm and an oxygen concentration of 4%. The results indicate that the original second-harmonic signals exhibit pronounced RAM-induced asymmetry, with an average LPIA of 18.07°, whereas after MHCD processing, the waveform symmetry is significantly improved and the LPIA is reduced to 8.56°. Furthermore, concentration discrimination experiments are performed on seven groups of vials with an oxygen concentration gradient of 2%. The second-harmonic integrated values extracted via quadrature demodulation are used for classification. The experimental results demonstrate that, while preserving the overall linearity of the detection results, the MHCD algorithm markedly increases the TPR for vials with low oxygen content and small concentration differences by 6–11%, while effectively reducing the FPR by 4–9% and the FNR by 0.5–7%. These results confirm the practical applicability of the MHCD algorithm on a physical detection platform and provide a technical foundation for enhancing sensitivity and discrimination reliability in pharmaceutical industrial online inspection lines. Furthermore, the MHCD algorithm was originally developed to suppress residual amplitude modulation by operating directly on demodulated harmonic components. Therefore, its applicability is not limited to TDLAS systems and it may also be extended to other modulation–demodulation optical sensing techniques that are sensitive to intensity or phase variations, such as photoacoustic spectroscopy, fiber-optic sensing systems and Pound–Drever–Hall technology.

## Figures and Tables

**Table 1:** Average RMSE between the reconstructed signals and the original signals.

Original Signal	1*f* Signal	2*f* Signal	3*f* Signal
Mean RMSE	0.002	0.009	0.0081

**Table 2:** The average RMSE index of the MHCD algorithm and comparative algorithm.

Mean RMSE	EMD	SG	FFT	MHCD
**Gaussian Noise**	0.0390	0.0387	0.0352	**0.0096**
**Baseline Drift**	0.0410	0.0451	0.0399	**0.0127**

**Table 3:** Comparison of the true positive rate and false positive rate before and after applying the MHCD algorithm.

Oxygen Concentration	Without MHCD			MHCD		
	TPR	FPR	FNR	TPR	FPR	FNR
0%	86.5%	13.5%	-	94%	6%	-
2%	68.5%	21.5%	10%	79.5%	12.5%	8%
4%	82.5%	4.5%	13%	93.5%	0.5%	6%
6%	84.5%	9%	6.5%	95%	1.5%	3.5%
8%	79%	17%	4%	85%	10.5%	4.5%
10%	82.5%	11%	6.5%	88.5%	7%	4.5%
12%	91%	-	9%	97.5%	-	2.5%

## Data Availability

The data that support the findings of this study are available on request from the corresponding author. The data are not publicly available due to privacy concerns.
